# Particulate Matter and Cardiovascular Disease: Researchers Turn an Eye toward Microvascular Changes

**DOI:** 10.1289/ehp.121-a282

**Published:** 2013-09-01

**Authors:** Julia R. Barrett

**Affiliations:** Julia R. Barrett, MS, ELS, a Madison, WI–based science writer and editor, has written for *EHP* since 1996. She is a member of the National Association of Science Writers and the Board of Editors in the Life Sciences.

Particulate matter (PM) has been consistently associated with cardiovascular disease development and progression,^1^^,^^2^ and is believed to contribute to development either indirectly through the autonomic nervous system or inflammatory responses, or directly via entry into systemic circulation and subsequent damage to blood vessels.^3^ However, it’s unclear whether changes in the microcirculation—the small veins (venules) and arteries (arterioles) that compose the majority of the circulatory system—might also contribute.^4^ A new study in *EHP* explores the impact of PM on small blood vessels by studying the retina.^5^

“Researchers suspect that air pollution may cause heart disease, in part, by limiting the blood vessels’ ability to bring blood to the heart. This hypothesis has been difficult to test since looking at the very small blood vessels in people’s hearts is challenging,” says Sara Adar, an assistant professor of epidemiology at the University of Michigan, Ann Arbor, who was not involved in the current study. “By using photographs of the tiny, hair-like blood vessels in people’s eyes, researchers are able to get a direct look at how air pollution may affect other very small blood vessels in the body like those that bring blood to our hearts.”

Adar and her colleagues used this approach in a previous analysis of data from the Multi-Ethnic Study of Atherosclerosis (MESA), a multicenter prospective investigation of cardiovascular disease.^6^ They found that both short- and long-term exposure to elevated levels of fine PM was associated with narrowing of the arterioles and widening of the venules, measured as central retinal arteriolar equivalents (CRAE) and central retinal venular equivalents (CRVE), respectively.^5^

Extending that approach to a younger and healthier cohort, investigators in the current study recruited 84 individuals aged 22–63 years old with no history of cardiovascular disease or diabetes. The participants, all of whom worked at the Flemish Institute for Technological Research (VITO) in Mol, Belgium, completed up to three clinical visits and answered questionnaires about current health, lifestyle factors, and time spent in traffic in the preceding 24 hours. Study visits included photography of the fundus (interior surface) of the right eye for each participant as well as blood pressure and heart rate measurements for participants who completed two or three visits. An air monitoring station within 10 km of the institute provided coarse PM and black carbon exposure data at 2, 4, 6, 24, and up to 48 hours prior to each visit.

**Figure 1:**
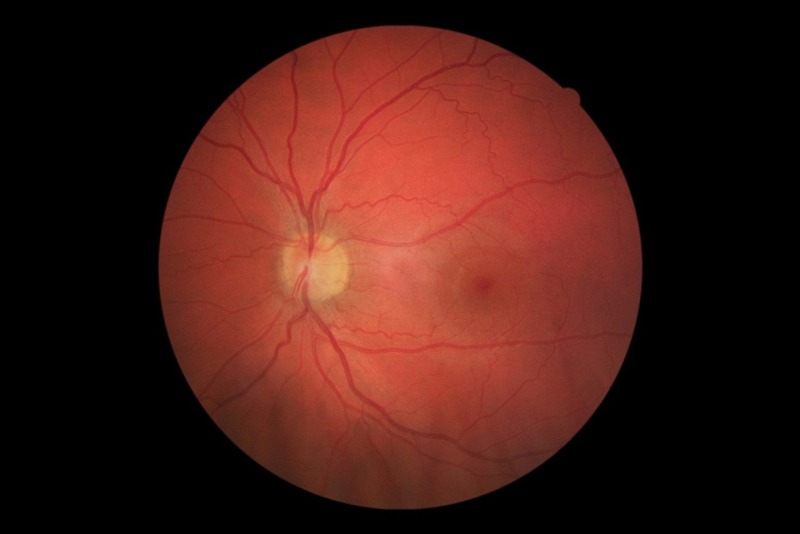
Pollution-related changes in the tiny blood vessels of the retina could offer insights into potential impacts on cardiovascular disease risk. © Tijs Louwies

During the course of the study (January to May 2012), observed CRVE did not change significantly, but decreases in CRAE were measured in association with higher exposures to coarse PM_10_ and black carbon. Associations remained significant in multiple statistical analyses, “no matter how we looked at the data,” says coauthor Luc Int Panis, research program coordinator at VITO. “We are convinced that this is a robust conclusion.” CRAE and CRVE were both associated with cardiovascular disease in other studies, although it is unclear whether they trigger the disease process or simply arise from it.

Int Panis notes the authors are not implying that the observed association has any immediate clinical implications. But he adds that the finding is consistent with downstream effects of air pollution that are already known to lead to atherosclerosis.

Both Int Panis and Adar note the “repeated measurements” design as a strength of the study. By collecting multiple (i.e., repeated) measurements on the same people over time, the authors were able to estimate the impacts of day-to-day fluctuations in pollution on individuals free of confounding by characteristics that vary among people, Adar explains.

The estimated changes in CRAE were about three times larger than those associated with similar levels of air pollution in the MESA analysis. However, the authors of the current study suggest that the younger and healthier study population may have had blood vessels that were better able to adapt to changing pollution conditions.^5^ The current study also looked solely at short-term exposures (2–24 hours) versus the short- and long-term exposures (24 hours and 2 years) evaluated in the MESA analysis. The researchers found no evidence of a threshold below which changes were not seen, consistent with the MESA analysis and other studies.^2^^,^^6^ “This well-conducted study confirms our previously published findings from the MESA study, which has indicated that air pollution may affect the very small blood vessels in our body,” says Adar.

As in other studies, individual exposure data were not available, raising the possibility of exposure misclassification. Further, the participants were not representative of the general population, so the results may not be broadly applicable. However, the investigators have already begun followup research with wearable air-monitoring devices, Global Positioning System devices, and a more diverse study population.
